# Resource Efficient Screening for Primary Prevention of Coronary Heart Disease: A Proof‐of‐Concept Test in the MESA Cohort

**DOI:** 10.1161/JAHA.124.038504

**Published:** 2025-03-21

**Authors:** Eva Hagberg, Elias Björnson, Martin Adiels, Anders Gummesson, Matthew Allison, Bledar Daka, Göran Bergström

**Affiliations:** ^1^ Department of Molecular and Clinical Medicine Institute of Medicine, Sahlgrenska Academy, Gothenburg University Gothenburg Sweden; ^2^ Department of Clinical Physiology Region Västra Götaland, Sahlgrenska University Hospital Gothenburg Sweden; ^3^ School of Public Health and Community Medicine Institute of Medicine, Sahlgrenska Academy, Gothenburg University Gothenburg Sweden; ^4^ Department of Clinical Genetics and Genomics Region Västra Götaland, Sahlgrenska University Hospital Gothenburg Sweden; ^5^ Department of Family Medicine University of California San Diego La Jolla CA USA; ^6^ Family Medicine, School of Public Health and Community Medicine Institute of Medicine, Sahlgrenska Academy, Gothenburg University Gothenburg Sweden

**Keywords:** coronary artery calcification score, coronary atherosclerosis, lipid‐lowering therapy, risk prediction tool, self‐reported data, Cardiovascular Disease

## Abstract

**Background:**

The best use of cardiac imaging to guide preventive coronary heart disease (CHD) treatment is debated. Current guidelines recommend the pooled cohort equation, followed by computed tomography for coronary artery calcification (CAC) assessment. We evaluated if this approach could be simplified using a self‐report risk algorithm instead of the pooled cohort equation.

**Methods:**

A gradient boosting machine model was trained on self‐reported factors to calculate the probability of a high CAC score (≥100). This model was part of a self‐report‐based CHD preventive strategy with 3 steps: (1) calculate the probability of having a high CAC; (2) perform computed tomography for high‐risk individuals; and (3) assign treatment eligibility with lipid‐lowering therapy if CAC score exceeds a designated threshold. This strategy was tested using data from the MESA (Multi‐Ethnic Study of Atherosclerosis) cohort (n=4564) and compared with guidelines recommending CAC scanning for intermediate‐risk individuals (pooled cohort equation, 7.5% to <20%) by evaluating CHD events over 10‐year follow‐up in the group defined as treatment eligible by either strategy.

**Results:**

The pooled cohort equation identified 33% of the MESA population as eligible for a CAC scan and 19% as treatment eligible, capturing 48% of all CHD events (103 of 216). The self‐report strategy identified 56% of CHD events (120 of 216; *P*=0.02) with the same number of CAC scans and treatments but required health care visits for only 33% of the population.

**Conclusions:**

A self‐report screening strategy, combined with CAC scoring, is more resource efficient and better discriminates high‐risk individuals suitable for lipid‐lowering therapy compared with current guidelines.

**Nonstandard Abbreviations and Acronyms**
CACS: coronary artery calcification score
MESA: Multi‐Ethnic Study of Atherosclerosis
PCE: pooled cohort equation
SCAPIS: Swedish CardioPulmonary BioImage Study

**Clinical Perspective**

**What Is New?**

- We developed a self‐report risk algorithm that effectively predicts the likelihood of coronary artery calcification and identifies individuals at high risk for coronary heart disease.
- The self‐report tool, combined with targeted coronary artery calcification scoring, offers a more resource‐efficient approach to identifying individuals at high risk for coronary heart disease, compared with current guidelines.

**What Are the Clinical Implications?**

- This self‐report strategy could complement current population and high‐risk–based preventive strategies, reducing the need for clinical visits to perform risk assessments.
- This strategy focuses resources on the ones most likely to benefit from lipid‐lowering therapy and optimize the use of cardiac imaging.

There is an ongoing debate around the best use of cardiac imaging in guiding primary preventive treatment of coronary heart disease (CHD).^1^ Currently, most guidelines recommend first assessing CHD risk with a traditional risk score, such as the pooled cohort equation (PCE), and then refining the risk for individuals at intermediate risk using computed tomography (CT) for coronary artery calcification (CAC).^2^, ^3^ However, this strategy is resource intensive as it requires an initial health care visit to calculate PCE.

We recently developed a self‐report prescreening test to identify individuals with a high risk of having moderate‐to‐severe coronary atherosclerosis (CAC score [CACS]≥100).^4^ We showed that a model with self‐reported variables performed as well as a model that also incorporated clinical variables (blood pressure, blood lipids, and blood glucose), in finding individuals with CACS ≥100.^4^ Importantly, self‐report prediction tools have been tested earlier^5^, ^6^ and are a potentially attractive component of future CHD screening strategies because they identify individuals at high cardiovascular risk without the need for an initial health care visit. As such, this strategy, when used in combination with CAC measurement, may result in a cost‐effective way of identifying and treating individuals at the highest risk.

We tested this strategy using data from the MESA (Multi‐Ethnic Study of Atherosclerosis) and comparing the self‐report strategy, combined with CAC measurements, with the current North American guidelines,^3^ which use the PCE with or without CAC scoring. Our primary hypothesis was that screening based on self‐report, in combination with CAC imaging, would use fewer initial health care visits and perform equally well as the current North American guidelines in identifying an incident CHD event during 10 years of follow‐up.

## METHODS

### Population

The MESA is a prospective observational study including individuals aged 45 to 84 years and without known clinical cardiovascular disease at the beginning of the study and with available information on CHD events during a follow‐up of >10 years. The initial examinations were conducted from 2000 to 2002 at 6 field centers. Detailed information about the study design, recruitment methods, examination components, and data collection can be found on the official MESA website: https://www.mesa‐nhlbi.org. MESA received approval from the institutional review boards at each participating center, and all participants provided written informed consent. The present study was also approved by the Swedish Ethical Review Authority (number 2021‐04030).

We excluded individuals aged >75 years, individuals with diabetes, and those with prior lipid‐lowering therapy. These exclusion criteria helped establish a study population where the PCE risk algorithm performs at its best (below the age of 75 years^3^) and the participants are treatment naive. We also excluded individuals with diabetes because they are at high risk and should receive lipid‐lowering therapy regardless of CACS.^3^, ^7^

### Risk Factors

We used available data on traditional risk factors to calculate the PCE risk score.^3^ Input data (8 factors) included age, sex, race, current smoking status, preexisting hypertensive medication, systolic blood pressure, high‐density lipoprotein cholesterol, and total cholesterol (Table 1).

**Table 1:** Factors in the Self‐Report Model (10 Factors), the PCE (8 Factors), and Their Measurement Scales

Category	Factor	Self‐report (scale)	PCE (scale)
Demographics	Sex	Binary	Binary
	Age	Continuous	Continuous
	Race	Categorical	Categorical
Anthropometry	Body weight	Continuous	N/A
	Body weight at age 20 y	Continuous	N/A
	Body height	Continuous	N/A
Smoking status	Cigarette pack‐years	Continuous	N/A
	Smoking duration	Continuous	N/A
	Current smoking	N/A	Binary
Health condition*	Antihypertensive medication	Binary	Binary
Blood tests	Total cholesterol	N/A	Continuous
	HDL cholesterol	N/A	Continuous
Clinical assessment	Systolic blood pressure	N/A	Continuous
Family history	Family history of myocardial infarction	Binary	N/A

HDL indicates high‐density lipoprotein; N/A, not applicable; and PCE, pooled cohort equation.

* Individuals with diabetes and/or lipid‐lowering therapy were excluded.

The self‐report model was trained on risk factors readily available without a clinical visit.^4^ Input variables were age, sex, race, body weight, body weight at 20 years of age, height, cigarette pack‐years, smoking duration, preexisting hypertensive medication, and family history of CHD (Table 1).

In MESA, demographic data, diabetes status, use of hypertensive medication, body weight at 20 years of age, current smoking status, cigarette pack‐years, smoking duration, and family history of CHD were collected from questionnaires. Systolic blood pressure was measured according to standardized procedures 3 times using an automated oscillometric device, with the reported average of the 2 last measurements.^8^ Body weight and height were measured according to standardized procedures.^8^ High‐density lipoprotein and total cholesterol levels were measured from blood samples.

CACS was measured in all individuals according to established protocols.^8^

Factors used in the pooled cohort equation (PCE), the self‐report strategy, and their measurement scales are shown in Table 1.

### Outcome Measures

CHD events were defined as myocardial infarction, resuscitated cardiac arrest, probable angina (if followed by a cardiac revascularization procedure), definite angina, and CHD death over a 10‐year follow‐up. Information on these CHD events was collected from medical records from hospitalizations, autopsy reports, and interviews with participants. In case of out‐of‐hospital death, information was gathered from interviews and questionnaires.^8^

In sensitivity analyses, we also tested other cardiovascular disease (CVD) outcomes, including the following: hard CHD (myocardial infarction, resuscitated cardiac arrest, and CHD death), hard CVD (myocardial infarction, resuscitated cardiac arrest, CHD death, and fatal and nonfatal stroke), and all CVD (myocardial infarction, resuscitated cardiac arrest, probable angina [if followed by a cardiac revascularization procedure], definite angina, CHD death, fatal and nonfatal stroke, as well as other atherosclerotic death and other CVD death).^8^ Full details of the MESA methods and adjudication procedures are available on the MESA website.

### Benchmarking the Self‐Report Strategy to Current Guidelines

Our aim was to compare a self‐report screening strategy for CHD events with the already established, guideline‐based PCE strategy.^3^ Both strategies use CAC measurements to refine the risk. We chose to define a successful screening strategy by how many of the individuals who later developed a CHD event that was deemed treatment eligible by the strategy. Our main comparison measure was therefore defined as the fraction of all CHD events that occurred, during 10‐year follow‐up, in individuals identified as treatment eligible by the self‐report and the PCE strategies. The strategies were also compared on how many initial clinical visits were needed in each strategy.

### Definition of Current Guidelines Using PCE and CACS

**Figure 1:**
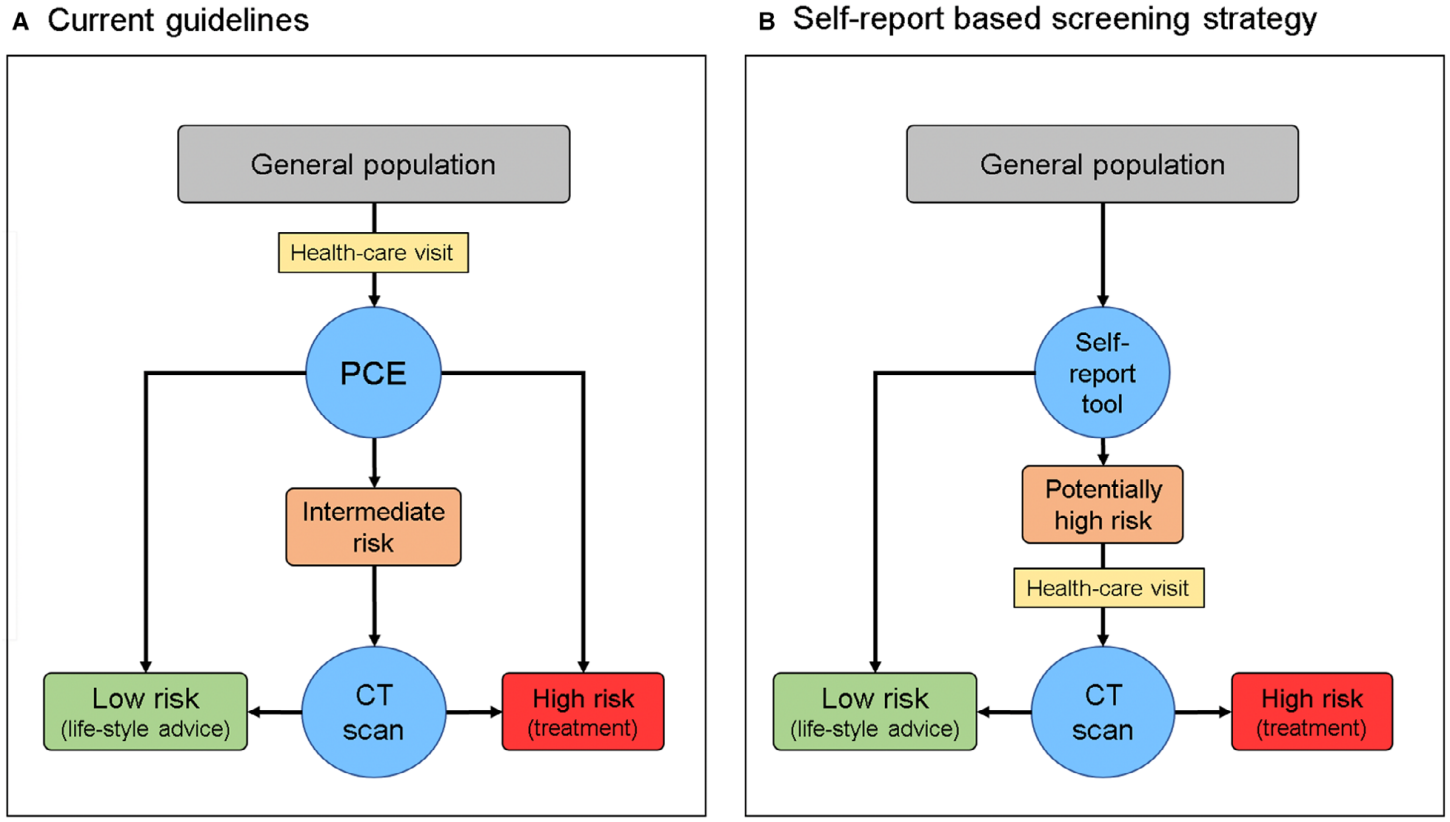
Outline of current guideline‐based strategy using PCE and CAC scoring vs the novel self‐report–based screening strategy. **A**, Current guidelines are based on the PCE with or without subsequent CT screening. If PCE‐estimated risk is high (≥20%), a person is eligible for lipid‐lowering therapy. If PCE‐estimated risk is intermediate (PCE ≥7.5% to <20%) and CACS is ≥100, treatment is also warranted. If these rules are followed, 852 of 4564 individuals (18.7%) are deemed eligible for lipid‐lowering therapy. **B**, In the self‐report screening strategy, we first estimate the risk of having a high CACS (≥100) based on self‐reported data. A CT scan for CAC scoring is then performed in those ranked at the highest risk. Treatment decision is then based on the measured CACS only. CAC indicates coronary artery calcification; CACS, CAC score; CT, computed tomography; and PCE, pooled cohort equation.

We used a simplified version of the current guidelines^3^ (Figure 1), which dictate preventive treatment with lipid‐lowering therapy if: PCE indicated high risk (≥20%) or if PCE indicated intermediate risk (≥7.5% to <20%) and data on CACS showed CAC ≥100. Low‐risk individuals (<5%) and individuals at borderline risk (5% to <7.5%) were not considered in need of treatment. We then calculated the number of CT scans and treatment‐eligible individuals resulting from this strategy. The same number of CT scans and treatments were then used as preestablished benchmarks in the self‐report screening strategy.

### Developing the Self‐Report Prediction Model With CAC Measurements of Those at High Risk

In our previous publication,^4^ 14 factors were used to develop a prediction tool for CACS ≥100. The prediction model was first adapted to the MESA cohort by selecting relevant variables from the MESA data set, resulting in a model based on a total of 9 variables (see Risk Factors [above]). Thus, 5 risk factors were removed from the previously published model: (1) questions on lipid‐lowering medication and (2) diabetes duration were redundant because of the exclusion criteria applied, measures of (3) waist and (4) hip were removed because they were deemed difficult to self‐report, and 1 question on (5) hypertension diagnosed by a physician was removed. To align with the MESA recruitment, which was stratified by race, we incorporated race as an additional variable, resulting in a set of 10 risk factors (Table 1).

Using these input variables, a gradient‐boosting model was trained to detect a CACS ≥100 and to output the predicted risk of having this outcome. The variable importance of the self‐report model was also calculated with gradient boosting. The individuals were then ranked according to their predicted risk of having CACS ≥100. The intention of the screening strategy is then to perform a CAC scan in individuals at high risk of having CACS ≥100. To make the 2 strategies comparable, the number of individuals who were intended for a CT scan was limited by the number of CT scans used in the guideline‐based PCE strategy. Individuals who qualified for a CT scan were then reranked according to their actual CACS data, and treatment eligibility was based *solely* on this rank order (and not on any other data). Again, the number of individuals who were treatment eligible was limited by the number of treated individuals using the guideline‐based PCE strategy to ensure fair comparisons.

### Validation of the Self‐Report Model in an External Cohort

The self‐report model was trained on MESA data, and its ability to identify individuals with CACS ≥100 was also quantified using this cohort. To control for the risk of overfitting, we also evaluated the performance of the model externally in the SCAPIS (Swedish CardioPulmonary Imaging Study).^9^ SCAPIS is a population‐based study with >30 000 participants focusing on the prevention of cardiac and pulmonary disease. SCAPIS has cross‐sectional data on similar risk factors and CACS as used in the development of the model in MESA. We validated the self‐report model on data from the Gothenburg site in SCAPIS (5968 participants).

### Converting the Self‐Report Model to a Useful Risk Assessment Tool in the Clinic

To benchmark the self‐report strategy against the current guideline‐supported strategy, the number of treatments and CT scans were, by definition, set equal. However, in a real‐world situation, the decision on whether to do a CT scan and whether to initiate lipid‐lowering therapy needs to be based on absolute thresholds. We therefore constructed examples of self‐report‐based strategies with fixed risk thresholds for CT screening and fixed CACS threshold for treatment eligibility. The threshold was selected on the basis of the results from the self‐report model benchmarked to the PCE strategy. We allowed for fewer number of people as eligible for lipid‐lowering therapy (the so‐called conservative strategy), a similar number (intermediate strategy), or a higher number of people (liberal strategy).

### Additional Analysis for CACS >0

In additional analysis, we used the same self‐report tool but instead to output the predicted risk of having CACS >0. We then used the same limitations for CAC scanning and treatment as in the primary analysis and investigated the ability to identify events.

### Statistical Analysis

All analyses were performed in R (version 4.1.3). For the self‐report model, we used the gradient boosting machine algorithm (gbm package in R, which is a decision‐tree–based machine‐learning model algorithm) to predict a CACS≥100. The model was trained using default settings provided in the gbm package without hyperparameter tuning. Specifically, number of trees were 100, learning rate (shrinkage) was 0.1, tree depth was 1, bag fraction was 0.5, and no stopping rule was used. Five‐fold cross‐validation was used during the model training procedure. To examine whether it was overtrained, a version of the model was trained on half of the MESA cohort and validated on the other half of the cohort. In addition, the model was externally validated in the SCAPIS cohort (data from the Gothenburg site). Importantly, the model was at no point trained to detect cardiovascular events. For comparisons between the event rates of the self‐report model and the PCE, we used the McNemar test function to assess whether the difference in proportions was statistically significant. The area under the receiver operating characteristic curve was calculated using the pROC package. We further investigated the difference in treatment eligibility in different PCE and CACS categories. We followed the Transparent Reporting of a Multivariable Prediction Model for Individual Prognosis Or Diagnosis‐ Artificial Intelligence (TRIPOD‐AI) protocol for transparent reporting.^10^ Although no formal statistical protocol was prespecified, all steps were documented. The analytical code is available on request.

## RESULTS

### Population

**Figure 2:**
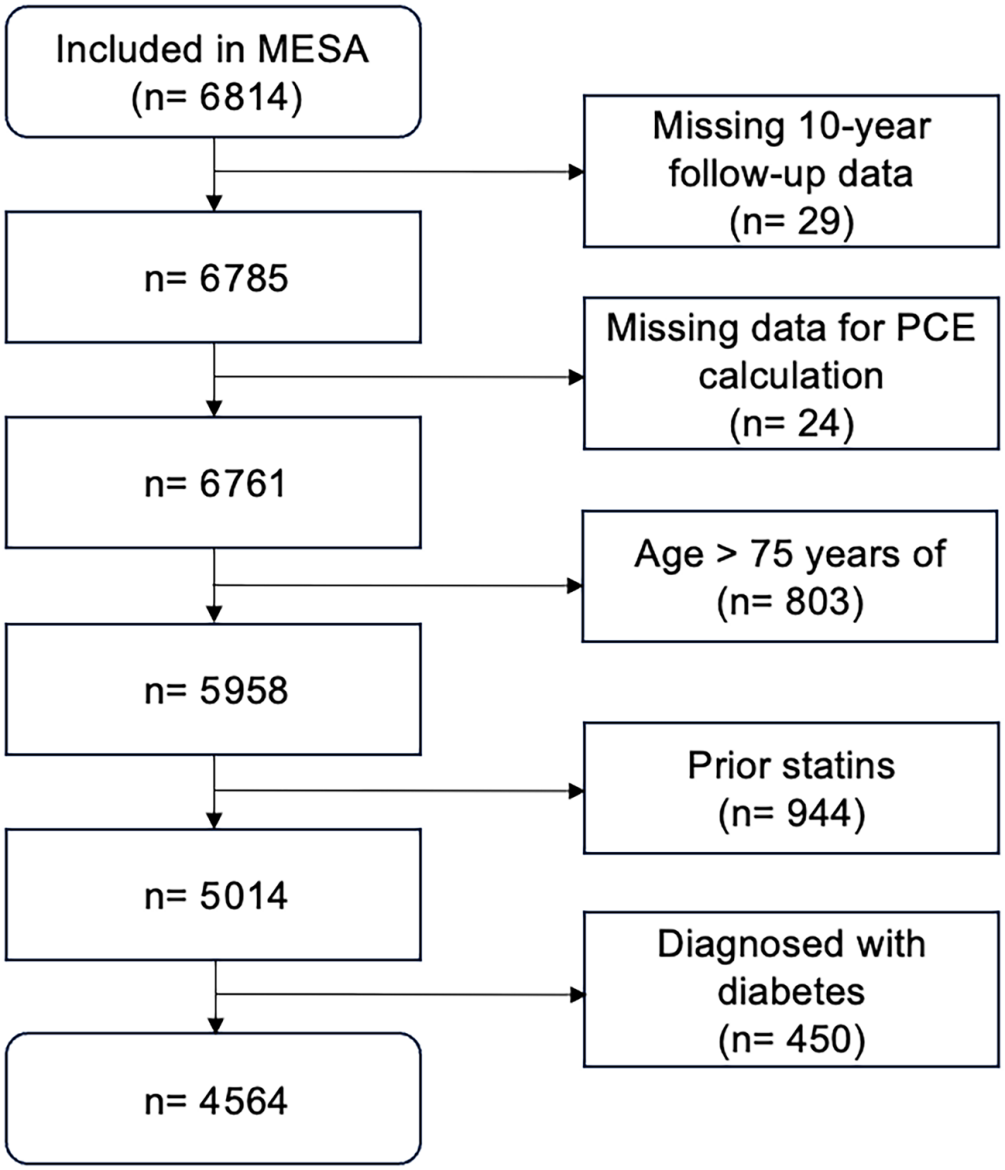
Flow diagram of the study inclusion. Individuals aged >75 years were excluded to optimize the performance of the PCE‐based strategy. Individuals with already ongoing lipid‐lowering therapy or having diabetes were excluded because the model will not change their need for lipid‐lowering therapy. MESA indicates Multi‐Ethnic Study of Atherosclerosis; and PCE, pooled cohort equation.

The data used in this study are available, and researchers can request access at http://www.mesa‐nhlbi.org. The MESA cohort includes a total of 6814 participants. We excluded 29 of these because of missing 10‐year follow‐up data. Another 24 subjects were excluded on the basis of missing data for the calculation of PCE risk. Another 803 subjects were excluded because of age >75 years (PCE performs best below age 75 years^3^). A further 944 subjects were excluded because of already ongoing lipid‐lowering medication, and last, 450 individuals were excluded because of established diabetes (which by itself is an indication for lipid‐lowering therapy; Figure 2). These exclusion criteria resulted in a set of 4564 individuals with a mean age of 59 years, of which 46.5% were men, nearly 40% were White, 27% were Black, 22% were Hispanic or Latino, and 12% were Chinese American (Table 2). The average PCE risk was 9%, with 56% classified as low, 33% as intermediate, and 10.5% as high cardiovascular risk by PCE.

**Table 2:** Baseline Characteristics of the 4564 Included Individuals

Characteristic	Value
Sex, N (%) (male/female)	2121/2443 (46.5/53.5)
Age, y	59.0±8.75
Race or ethnicity, N (%)	
White	1792 (39.3)
Chinese	563 (12.3)
Black	1212 (26.6)
Hispanic or Latino	997 (21.8)
Body weight, kg	78.4±17.2
Waist, cm	96.8±14.4
Total cholesterol, mg/dL	198±35.2
LDL cholesterol, mg/dL	121±31
HDL cholesterol, mg/dL	51.4±15.1
Triglycerides, mg/dL	128±86
Systolic blood pressure, mm Hg	124±20.4
CACS	86.6±286
CACS=0, N (%)	2691 (59)
CACS 1–99, N (%)	1125 (25)
CACS ≥100, N (%)	746 (16.3)
PCE, %	9.0±8.0
PCE risk, N (%)	
<7.5% (Low)	2566 (56.2)
≥7.5% to <20% (Intermediate)	1519 (33.3)
≥20% (High)	479 (10.5)
All CHD events, N (%)	216 (4.7)
Hard CHD events, N (%)	134 (2.9)
All CVD events, N (%)	304 (6.7)
Hard CVD events, N (%)	236 (5.2)

Data are given as mean±SD unless otherwise indicated. All CHD events: myocardial infarction (MI), resuscitated cardiac arrest, probable angina (if followed by a cardiac revascularization procedure), definite angina, and CHD death. Hard CHD events: MI, resuscitated cardiac arrest, and CHD death. All CVD events: MI, resuscitated cardiac arrest, probable angina, definite angina, CHD death, fatal and nonfatal stroke, atherosclerotic death, and CVD death. Hard CVD events: MI, resuscitated cardiac arrest, CHD death, and fatal and nonfatal stroke. CACS indicates coronary artery calcification score; CHD, coronary heart disease; CVD, cardiovascular disease; HDL, high‐density lipoprotein; LDL, low‐density lipoprotein; and PCE, pooled cohort equation.

### Predictors of CAC

The variables used in the simplified PCE strategy and the self‐report tool are shown in Table 1. The variable‐importance analysis from the self‐report model, shown in Figure S1, indicates that age, cigarette pack‐years, and sex were the most significant self‐reported predictors in this model, followed by body weight at age 20 years, race, and a family history of CHD.

### Number of CAC Scans and Number of Individuals Eligible for Lipid‐Lowering Therapy

**Figure 3:**
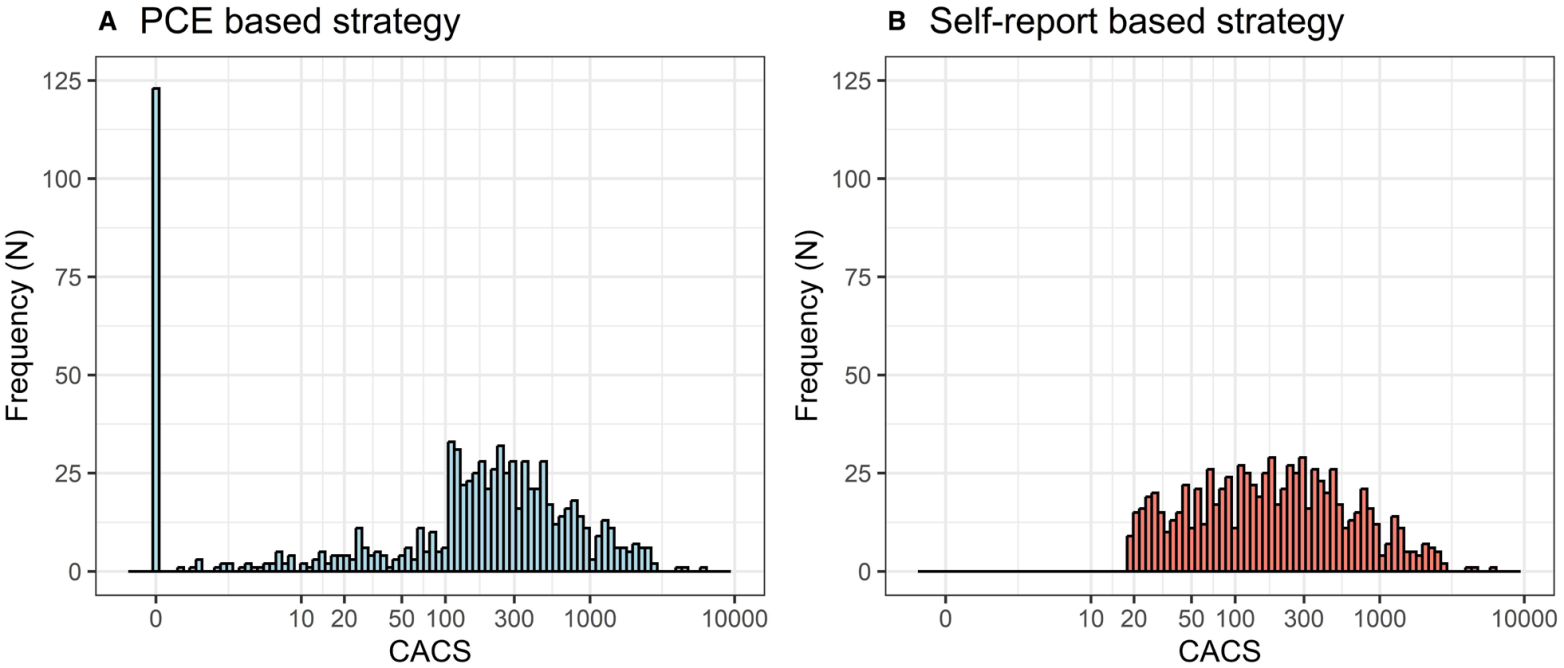
CACS distribution in groups eligible for lipid‐lowering therapy as defined by either strategy. **A**, CACS distribution in the group deemed eligible for lipid‐lowering therapy as defined by the current guideline‐based PCE strategy. In the subgroup deemed eligible for lipid‐lowering therapy based on a PCE >20%, 123 individuals had a CACS score of 0. **B**, CACS distribution in the group deemed eligible for lipid‐lowering therapy by the self‐report strategy. The lowest CACS score in the self‐report group was 18. CACS indicates coronary artery calcification score; and PCE, pooled cohort equation.

The PCE‐based screening strategy (Figure 2A) resulted in 479 (10.5%) individuals classified at high risk (PCE ≥20%) and deemed eligible for lipid‐lowering therapy. In addition, 1519 individuals (33.3%) were of intermediate risk and thus eligible for a CT scan. The CAC data for individuals at intermediate risk showed that 373 of them (24.5%) had CACS ≥100 and were therefore also deemed eligible for lipid‐lowering therapy (Figure 3A). Thus, the total fraction of the population deemed eligible for lipid‐lowering therapy using the simplified PCE strategy was 18.7%, or 852 individuals.

The self‐report–based screening strategy is outlined in Figure 2B. The number of CAC measurements and the number of participants eligible for lipid‐lowering therapy were matched to the PCE‐based strategy (ie, n=1519 and n=852 individuals, respectively). We first ranked the individuals for their predicted risk of having CACS ≥100 according to the self‐report algorithm. The 1519 individuals with the highest rank were selected for CAC measurement. In total, 68% (1034 of 1519 of those with high risk according to the self‐report model) had CACS >0, and 37% (557 individuals) had CACS ≥100. We then ranked these individuals based on their measured CACS and defined the 852 participants with the highest CACS as eligible for lipid‐lowering therapy (Figure 3B; lowest CACS among this group was 18).

### The Self‐Report Strategy Uses Fewer Health Care Resources

**Figure 4:**
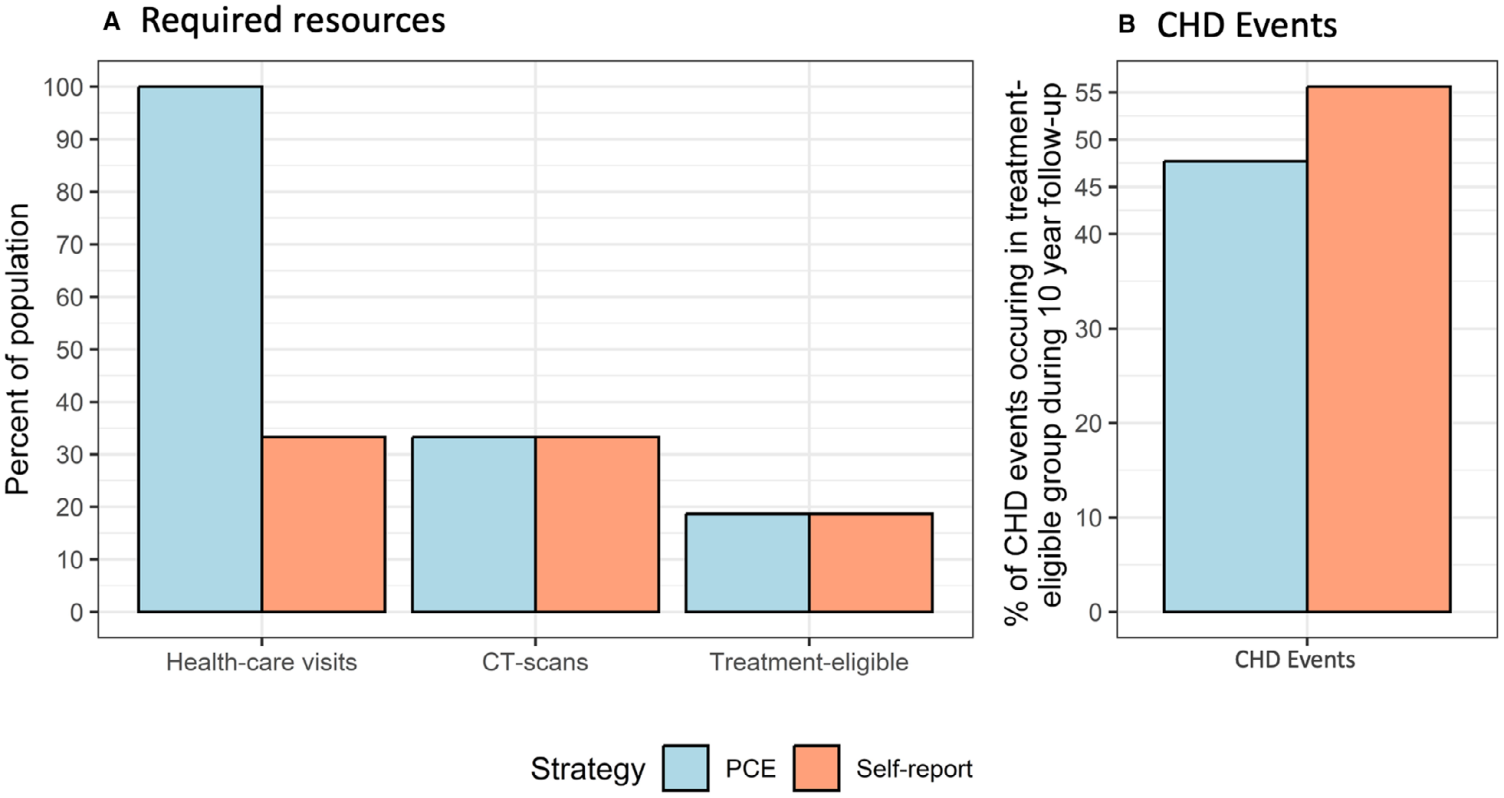
Health care resources required by the 2 strategies and their capacity to identify individuals who develop CHD. **A**, Health care resources required for the 2 screening strategies. The self‐report–based strategy requires fewer initial health care visits and, by design, the same number of CT scans and treatments. Note that the similarity in proportions of CT scans and treatments between strategies is intentional for comparison; however, the specific individuals identified being at high risk differ. **B**, The self‐report screening strategy shows a higher capacity to identify individuals who later develop CHD compared with the PCE‐based strategy (PCE strategy, 47.7%; self‐report strategy, 55.6%; *P* value for difference=0.017). CHD indicates coronary heart disease; CT, computed tomography; and PCE, pooled cohort equation.

To calculate the PCE risk score for an individual, blood pressure and blood cholesterol must be quantified, and thus a health care visit is required for 100% of the population (n=4564; Figure 4A). The final decision on treatment eligibility is then decided after all individuals with an intermediate PCE‐based risk have been CT scanned.

In contrast, the decision on eligibility for lipid‐lowering therapy in the self‐report strategy is solely based on the measured CACS of the individuals selected for a CT scan. The number of CT scans were, by definition, set as identical to the PCE‐based strategy (n=1519; 33.3% of the population). The number of treatments were also identical (n=852; 18.7%). Measuring blood pressure and blood lipids is initially technically nonmandatory for the 33.3% of the population selected for CT screening in the self‐report strategy; however, it is important for best decisions on intervention against modifiable risk factors. Thus, 33.3% of the population (n=1519), require a health care visit using the self‐report strategy (Figure 4A).

### The Self‐Report Strategy With CAC Scoring Identifies More Individuals Who Later Develop CHD

Using the PCE‐based screening strategy, 48% of all events (103 of 216 events) occurred in the group deemed eligible for lipid‐lowering therapy (Figure 4B). Using the self‐report screening strategy, significantly more individuals (*P*=0.02) who later experienced a CHD event were found in the treatment‐eligible group (120 of 216 events [56%]; Figure 4B). The age and sex distribution in the groups eligible for lipid‐lowering therapy were comparable for both strategies (self‐report strategy, 73% men and mean age of 68 years; PCE‐based strategy, 72% men and mean age of 68 years).

In the additional analysis of applying the self–report strategy to individuals with any detectable CAC (CACS >0), we identified 55% of CHD events.

### The Self‐Report Strategy Prioritizes Treatment to Subjects With Higher CACS and Higher CHD Event Rate, Compared With the PCE‐Based Strategy

In total, 852 individuals were eligible for lipid‐lowering therapy in the PCE‐based strategy. The CHD event rate in this group was 12% after 10 years of follow‐up. Treatment eligibility was mostly based on treating those with a PCE >20% (n=479 individuals, 56% of all treated), and the CHD event rate in this group was 10%. In the group of individuals with PCE >20%, 123 (26%) had a 0 CACS, and the event rate in this subgroup was considerably lower (4.1%). In total, 14% of those deemed treatment eligible in the PCE‐based strategy had 0 CACS (Figure 3A).

In the group of participants made eligible for lipid‐lowering therapy by the self‐report strategy (n=852), all individuals had a CACS >15 (Figure 3B), and the CHD event rate was 14% after 10 years of follow‐up. Table S1 illustrates how individuals are classified differently across PCE categories and CACS, highlighting the distinctions in eligibility and event rates between the 2 strategies.

### Model Performance and Sensitivity Analyses

When a version of the model was trained on half of the MESA cohort and evaluated on half of the cohort, the prediction accuracy for CACS ≥100 was not significantly different from the model using 5‐fold cross‐validation (Figure S2A), and the calibration curve demonstrates good model calibration (Figure S2B). When the model was evaluated in the external data set from SCAPIS, a slightly lower prediction accuracy (area under the receiver operating characteristic curve) for detection of CACS ≥100 was found (Figure S3A), However, a refitted model within the SCAPIS cohort showed a similarly lower accuracy, indicating that cohort‐specific attributes, and not overtraining, explained this finding. The model was well calibrated in SCAPIS, indicating external validity (Figure S3B).

In a first sensitivity analysis, the self‐report strategy was further benchmarked against a PCE‐based strategy where eligibility for lipid‐lowering therapy in the intermediate risk group was determined on the basis of age‐ and sex‐adjusted CACS (as opposed to an absolute CACS cutoff).^11^ In this scenario, 17% of the population was deemed treatment eligible (n=758 individuals). Using these criteria, the self‐report–based strategy still showed a higher ability to predict CHD events compared with the PCE strategy (51% compared with 42%; *P*=0.01).

In a second sensitivity analysis, we evaluated the performance of the self‐report strategy compared with the PCE‐based strategy across different sets of cardiovascular end points; in addition to using all CHD events as in the main analysis, we also evaluated (1) hard CHD events, (2) all CVD events, and (3) hard CVD events. A total of 46%, 46%, and 45% of all events for these 3 outcomes sets, respectively, occurred in the treatment‐eligible group identified by the PCE strategy. Corresponding numbers for the self‐report–based strategy were 52%, 50%, and 47%, respectively.

### Evaluating 3 Examples of Real‐World Self‐Report Strategies

**Figure 5:**
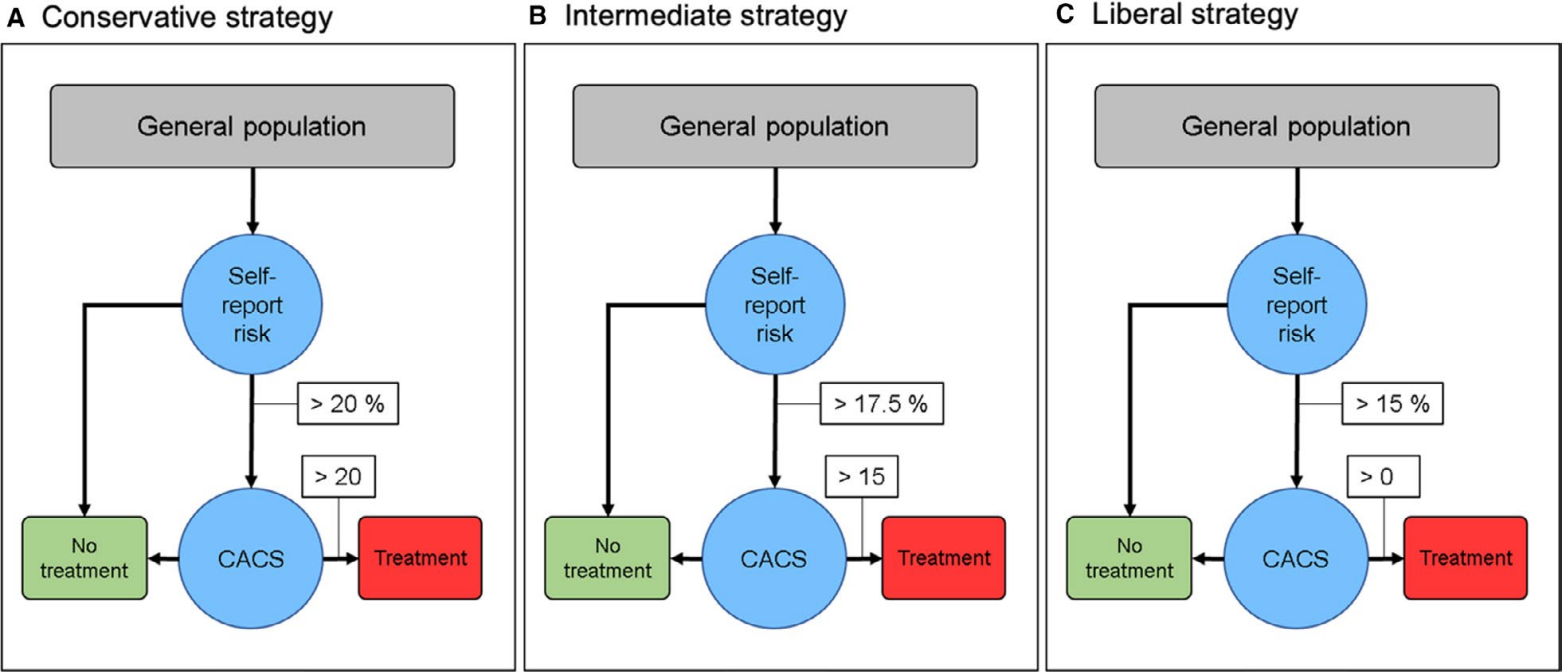
Three examples of real‐world screening scenarios using self‐report with fixed cutoffs. The risk cutoff for being eligible for CT screening and the CAC cutoff for treatment can be tuned according to preferences for screening/treatment availability. We selected strategies in which the risk of having a high CACS (≥100) based on self‐reported data varied between >15% and >20%. The CACS score at which lipid‐lowering therapy was recommended varied between >0 AU and >20 AU. This created a conservative (A), intermediate (B), and liberal (C) strategy when compared with our benchmarked model. The resulting number of health care visits, CT screenings, treatments, and event rate is presented in Figure 6. AU indicates Agatston unit; CAC, coronary artery calcification; CACS, CAC score; and CT, computed tomography.

**Figure 6:**
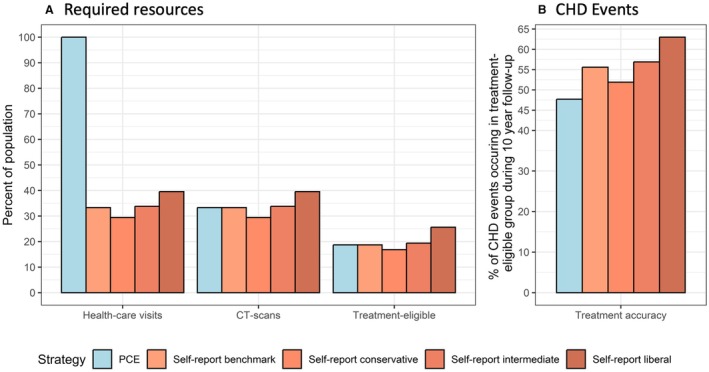
Resources required and treatment accuracy of all 5 screening strategies presented. **A**, Resources required for each screening strategy. The self‐report–based strategies require fewer health care visits and a comparable number of CT scans and treatments. **B**, The self‐report screening strategies show equal or greater ability to predict CHD events compared with the PCE‐based strategy (PCE strategy, 47.7%; self‐report conservative strategy, 51.9%; self‐report intermediate strategy, 56.9%; liberal strategy, 63.0%). CHD indicates coronary heart disease; CT, computed tomography; and PCE, pooled cohort equation.

Building on our initial model, we tested a total of 3 strategies using fixed risk thresholds for CT screening and a fixed CACS threshold for eligibility for lipid‐lowering therapy (Figure 5). These strategies allowed for fewer number of people as treatment eligible (the so‐called conservative strategy), a similar number (intermediate strategy), or a higher number of people (liberal strategy). All strategies showed equal or higher accuracy in identifying high‐risk individuals compared with the PCE‐based strategy (*P* values for difference in number of CHD events occurring in treatment‐eligible group of *P*=0.21, *P*<0.01, and *P*<0.01 for conservative, intermediate, and liberal strategies, respectively). All strategies used fewer initial health care resources (Figure 6).

## DISCUSSION

Developing new strategies for CHD prevention is crucial, given the stagnant mortality rates related to coronary heart disease^12^ and the fact that variations in health care accessibility lead to differing guideline implementation across societal groups.^13^ In the current report, we presented data showing that a novel strategy to identify individuals at high risk of developing CHD using self‐reported data on cardiovascular risk, combined with CAC measurements, is superior to the current guideline‐based strategy in which individuals at intermediate risk according to the PCE receive a CT scan to measure CACS. Not only is the self‐report strategy significantly better in predicting CHD events, it likely saves resources by using substantially fewer initial health care visits. Furthermore, the self‐report questionnaire is well suited as a web‐based tool that is a resource‐effective way of reaching individuals across different societal groups. The self‐report model uses established CHD risk factors, and our variable importance analysis revealed that continuous smoking measures, such as pack‐years, provided strong predictive value. Additionally, weight at age 20 years showed a stronger correlation with a CACS≥100 compared with current weight, which is consistent with prior research findings.^14^

Most people do not know their current blood pressure and lipid profile, and cannot calculate their CHD risk using PCE or equivalent risk calculators without the need for a health care visit. The need for a health care visit also results in a relatively high practical barrier to CHD risk estimation. This may partly explain why high‐risk individuals are currently undertreated in the United States and in the United Kingdom.^13^ A web‐based self‐report tool has the advantage of potentially circumventing this problem given that it can be used freely, and internet access is widely available. In a real‐world setting, one of the advantages with the self‐report tool could therefore be its ability to reach a higher proportion of high‐risk individuals because of its ease of use. Self‐report prediction tools have been successfully tested earlier,^5^, ^6^ but not incorporated in a preventive strategy using CACS.

In MESA, CT was used for cardiac imaging, and we therefore used CACS for risk assessment. An alternative would be to use coronary CT angiography for screening, which would provide a more comprehensive assessment of coronary disease.^15^ Although CT angiography may improve risk stratification, it involves higher radiation exposure and requires the administration of contrast agents, which may not be suitable for all individuals. Our findings demonstrate that CAC scoring is a valuable tool for initial risk stratification, effectively identifying patients who may be at risk of future cardiovascular events. CAC scoring involves limited radiation exposure, and if expanded to scanning 40% of the high‐risk population and treating 25%, this could reach as many as 63% of all individuals experiencing an event.

CAC scoring is highly specific for coronary artery disease^16^ and well established in its ability to identify individuals at elevated risk of future coronary events, as demonstrated in several observational trials.^17^, ^18^ A CACS ≥100 is associated with an increased risk of CHD events in individuals aged 40 to 75 years across sex and racial groups.^19^ However, this threshold might not imply a similar increase in risk in younger and older individuals, and further data are needed for these subgroups.^20^

Ongoing randomized controlled trials aim to establish more robust evidence on the benefits of preventive treatment guided by CAC scoring in reducing cardiovascular events.^21^, ^22^ Propensity score–matched studies using registry data suggest a great value of lipid lowering in individuals with a high CACS.^23^ Current guidelines recommend using CAC scoring for risk stratification in intermediate risk individuals based on either PCE or the European Systematic COronary Risk Evaluation 2.^3^, ^7^ However, there is no established cost‐effectiveness, and the current strategies are not optimized toward the use of CACS.^24^

The strategy presented in this article represents an extension to our previous work^4^ to optimize the use of CAC scanning for risk estimation. The first step in this strategy focuses on self‐report to identify individuals with the highest probability of having CACS ≥100. These high‐risk individuals are then selected for CAC screening, but treatment recommendations are ultimately based on the actual measured level of CACS. In contrast to this strategy, the current guidelines support measuring CACS in individuals at intermediate risk of an incident CHD event.^3^ From the current results, it appears that the guideline‐based strategies do not maximize the value of the CAC measurement and result in many negative scans. Indeed, we show that only 25% of the individuals at intermediate risk have CACS ≥100.

In addition, the guideline‐based strategy allocates all high‐risk individuals (PCE >20%) to lipid‐lowering therapy. One reason for the lower treatment accuracy of the guideline‐based strategy compared with self‐report was that a considerable number of the high‐risk individuals had a 0 CACS (25.7%). This group had a low (4.1%) frequency of CHD events during the 10 years of follow‐up despite having high PCE. The self‐report–based strategy, in contrast, did not prioritize treatment for anyone with a 0 CACS (Table S1). Arguably, it may be correct to treat individuals with a PCE >20%, regardless of CACS, but the self‐report strategy evidently identified more individuals with higher 10‐year CHD risk. As such, the self‐report–based strategy increases the ability to identify individuals eligible for lipid‐lowering therapy and could be attributed to the fact that current guidelines allocate lipid‐lowering therapy to people with 0 CACS, something the self‐report–based strategy does not do.

In additional analysis, we applied the self‐report strategy to identify individuals with detectable CAC (ie, CACS >0) and found that a similar proportion of all CHD events were identified. This result is consistent with the findings from our primary analysis, showing that the self‐report model is robust and can be applied across different CACS thresholds.

### Clinical Implication

The presented screening strategy has several potential applications in real‐world clinical settings. The self‐report strategy effectively identified a substantial proportion of individuals with any detectable CAC and a significant subset with CACS ≥100, supporting its potential to guide early intervention decisions. We evaluated how different fixed cutoff values for CACS would impact the number of individuals eligible for treatment and investigated the resultant number of CHD events occurring in the treatment‐eligible groups. All strategies demonstrated equal or higher ability to identify high‐risk individuals compared with the guideline‐based PCE strategy while requiring fewer health care resources. Using a cutoff probability of 18% for having a CACS ≥100 in combination with lipid‐lowering therapy for everyone with a CACS >15, we managed to allocate lipid‐lowering therapy to individuals in which 57% of all future CHD events occurred. Health‐economic analyses are needed to determine the cost‐effectiveness of this novel strategy.

One of the key advantages of this approach is its ability to identify high‐risk individuals in their homes through a self‐report test, and in a real‐world clinical setting it could serve as a complementary method to current population and high‐risk–based preventive guidelines. Combining this screening strategy with current practice can potentially enhance adherence to preventive measures, engage individuals in shared decision‐making, and improve overall cardiovascular outcomes. Implementing this strategy in clinical practice may also face challenges, including variable participant rate for remote users and concerns about transparency and interpretability of machine learning models. Additionally, clinical adoption would require adjustments to existing infrastructure and workflow. Addressing these challenges through robust validation, user‐centered design to improve accessibility, and transparent communication of model functionality would be important for clinical implementation.

### Limitations

First, we have used prospectively collected data from the MESA cohort to conduct a post hoc secondary analysis of our screening strategy. A prospective study is still needed to establish firm evidence. Second, it is conceivable that overtraining of our models may explain a higher treatment accuracy for the self‐report strategy. However, it is important that the self‐report model, unlike PCE, was never trained to detect CHD events and thus could not have been overtrained in this regard. Also, a version of the model that was trained on half of the MESA cohort and evaluated on half of the cohort could detect CACS ≥100 equally well as the model trained using 5‐fold cross‐validation. Furthermore, we evaluated the model externally in the SCAPIS cohort. A slightly lower area under the receiver operating characteristic curve was found; however, a refitted model within the SCAPIS cohort showed a similarly lower accuracy, indicating that cohort‐specific attributes, and not overtraining, were the cause of this. The model was found to be well calibrated in SCAPIS, indicating external validity. Third, the proposed screening strategy is compared with a simplified PCE/CACS approach to match the number of CT scans and treatments. To streamline the identification of high‐risk individuals, we used a model based on excluding individuals with a PCE of 5% to 7.5% from treatment. We recognize that some individuals with PCE scores of 5% to 7.5% may still qualify for treatment based on risk‐enhancing factors or CAC testing. Future studies could explore broader thresholds and additional clinical factors.

We acknowledge that the choice to perform CAC imaging is a more complex process influenced by both clinical and practical considerations and individual patient preferences. Last, body weight at age 20 years was self‐reported, which may introduce recall bias. However, a correlation of 0.82 with measured weights in previous studies suggests that this approximation is acceptable for large‐scale epidemiologic research.^25^

In conclusion, we showed that an easy‐to‐use self‐report–based CHD screening program coupled with targeted CT screening of coronary calcification in a select high‐risk population may be superior to a guideline‐based screening strategy in identifying high‐risk individuals suitable for preventive lipid‐lowering therapy.

## Sources of Funding

The study was financed by grants from the Swedish Heart Lung Foundation (20210383), the Swedish Research Council (2019‐01140), and the Swedish state under the agreement between the Swedish government and the county councils, the ALF agreement (Agreement on Medical Education and Research), and Avtal om Läkarutbildning och Forskning (ALFGBG‐718851). SCAPIS (Swedish CardioPulmonary BioImage Study) is supported by the Swedish Heart and Lung Foundation, the Knut and Alice Wallenberg Foundation, the Swedish Research Council and *Verket för Innovationssystem*, VINNOVA (Swedish Government Agency for Innovation Systems).

## Disclosures

None.

## Supporting information

Table S1
Figures S1–S3
