# Supplementary material for: Resource Efficient Screening for Primary Prevention of Coronary Heart Disease: A Proof‐of‐Concept Test in the MESA Cohort
Source: J Am Heart Assoc. 2025 Mar 21;14(7):e038504. doi: 10.1161/JAHA.124.038504 (PMC12132897; doi:10.1161/JAHA.124.038504)
Supplement: Supplementary file 1 — Table S1 Figures S1–S3 [file JAH3-14-e038504-s001.pdf]

Supplemental Material

Table S1. Comparison of eligibility based on the simplified PCE and the self-report strategy.

Categories include individuals not eligible for treatment by either model, "Neither", eligible by both models, eligible only by PCE, and eligible only by the self-report model. Event rates are provided for each subgroup. Note that the event risk in the "Eligible only by PCE" group with a CAC score of 0 was 4.1% which is the main reason for the higher identification rate of the self-report model.

Category	Neither (n=3453)	Eligible by both (n=593)	Eligible only by PCE (n=259)	Eligible only by the Self report (n=259)	p
PCE_category (n, %)					
< 5%	1902 (55.1)	0 (0.0)	0 (0.0)	22 (8.5)	<0.001
5 to 7.5%	591 (17.1)	0 (0.0)	0 (0.0)	51 (19.7)	
7.5 to 20%	960 (27.8)	301 (50.8)	72 (27.8)	186 (71.8)	
>20%	0 (0.0)	292 (49.2)	187 (72.2)	0 (0.0)	
CACS category (n, %)					<0.001
0	2568 (75.1)	0 (0.0)	123 (47.7) †	0 (0.0)	
1 to 99	739 (21.6)	76 (12.8)	60 (23.3)	217 (84.4)	
≥ 100	114 (3.3)	517 (87.2)	75 (29.1)	40 (15.6)	
CHD Events* (n,%)	79 (2.3)	86 (14.5)	17 (6.6)	34 (13.1)	<0.001

*CHD events were defined as myocardial infarction (MI), resuscitated cardiac arrest, probable angina (if followed by cardiac revascularization procedure), definite angina and CHD death over a 10-year follow-up. CHD: Coronary Heart Disease, CACS: Coronary Artery Calcium Score, PCE: Pooled Cohort Equation

†CHD event rate of 4.1%.

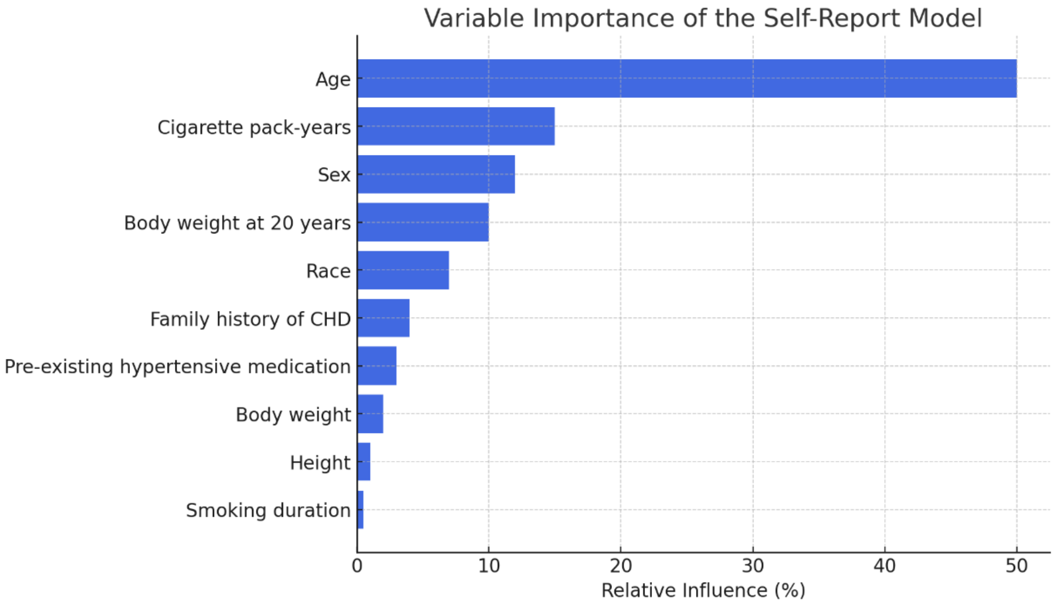

Figure S1. Relative influence (%) of different variables in predicting the likelihood of having a CAC score ≥100 using the self-report model. Age is the most influential factor, followed by cigarette pack years, sex, and body weight at 20 years.

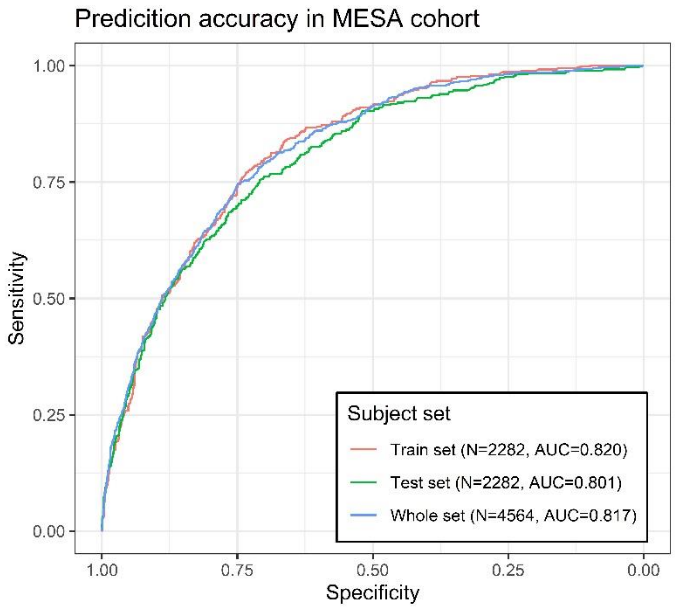

Figure S2. Prediction accuracy of the self-report model. The ability of the self-report model in identifying subjects with CACS≥ 100 was evaluated in the whole subject set (N=4564) using a model fitted by 5-fold cross-validation or using a model fitted in half of the population (train set, N=2282) and evaluated in the other half (test set, N=2282). The accuracy of the model was 0.80-0.82. The ROC-AUC values were not statistically significantly different from each other; indicating that the model was not over-trained.

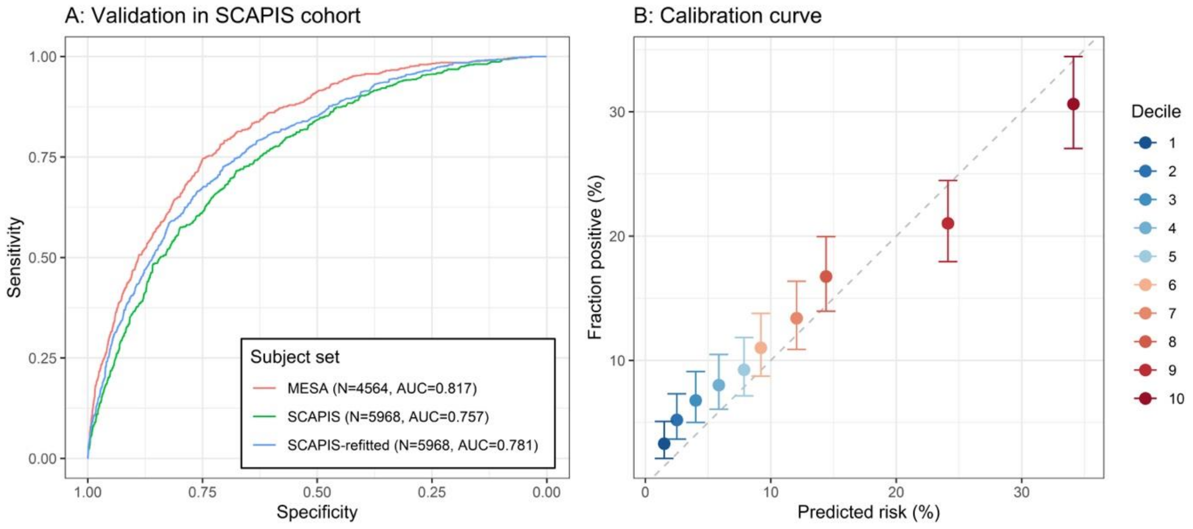

Figure S3. Prediction accuracy of the self-report model in the SCAPIS cohort. The ability of the self-report model in identifying subjects with CACS ≥ 100 was quantified in the MESA cohort and externally validated using data from the Gothenburg site of the SCAPIS cohort. The model was found to be less accurate in the SCAPIS cohort (AUC = 0.757), but well calibrated. To evaluate whether the lower accuracy could be explained by over-training in the MESA cohort, a re-fitted model was also constructed in SCAPIS, using the same nine variables and 5-fold cross-validation. The accuracy increased to 0.781 but was still lower than in the MESA cohort – indicating that over-fitting was not the predominant cause of the slightly lower accuracy in the SCAPIS cohort. Overall, the model is well calibrated and is usable in a population outside of MESA.
